# Supplementary material for: Relational graph convolutional networks: a closer look
Source: PeerJ Comput Sci. 2022 Nov 2;8:e1073. doi: 10.7717/peerj-cs.1073 (PMC9680895; doi:10.7717/peerj-cs.1073)
Supplement: Supplemental Information 1 [file peerj-cs-08-1073-s001.pdf]

Sources of third-party data

Node Classification

- `AIFB` from *Stephan Bloehdorn and York Sure. Kernel methods for mining instance data in ontologies.. In The Semantic Web, 6th International Semantic Web Conference, 2007.*
  Link to data:
  https://www.dropbox.com/sh/ldjd70yvnu9akxi/AAAam7SBr5KXLfjk-NVGQNWRa?dl=1
- `MUTAG` from *A. K. Debnath, R. L. Lopez de Compadre, G. Debnath, A. J.Shusterman, and C. Hansch. Structure-activity relationship of mutagenic aromatic and heteroaromatic nitro-compounds correlation with molecular orbital energies and hydrophobicity. J Med Chem,34:786–797, 1991.*
  Link to data:
  https://www.dropbox.com/sh/tburaaxij0a1vmy/AAAlD5ORzcMbF3YpoynOLGqwa?dl=1
- `BGS` from *de Vries, G.K.D. A fast approximation of the Weisfeiler-Lehman graph kernel for RDF data. In European Conference on Machine Learning and Principles and Practice of Knowledge Discovery in Databases, 2013.*
  Link to data:
  https://www.dropbox.com/sh/so1n0zc4zkel2mf/AACq3Ilckg1AAMfi2umI3MbGa?dl=1
- `AM` from *de Boer, V., Wielemaker, J., van Gent, J., Hildebrand, M., Isaac, A., van Ossenbruggen, J., Schreiber, G. Supporting linked data production for cultural heritageinstitutes: The amsterdam museum case study. In The Semantic Web: Research and Applications, 2012.*
  Link to data:
  https://www.dropbox.com/sh/5ys1lfw9c8padz0/AABEJChkUHkxrWfvXrgehOX5a?dl=1

Link Prediction

- `WN18` from *Antoine Bordes, Nicolas Usunier, Alberto Garcia-Duran , Jason Weston, and Oksana Yakhnenko. Translating embeddings for modeling multi-relational data. In Advances in Neural Information Processing Systems, 2013.*
  Link to data:
  https://www.dropbox.com/sh/egwgth011epusq7/AABWx1YWuEaMoumHDOknbCA9a?dl=1
- `FB–Toy` from *Daniel Ruffinelli, Samuel Broscheit, and Rainer Gemulla. You CAN teach an old dog new tricks! on training knowledge graph embeddings. In International Conference on Learning Representations, 2019.*
  Link to data:

https://www.dropbox.com/sh/5kv7xk4cj1md9zw/AADpaREEK9K5NX_Vb5eRcXuRa?dl=1
